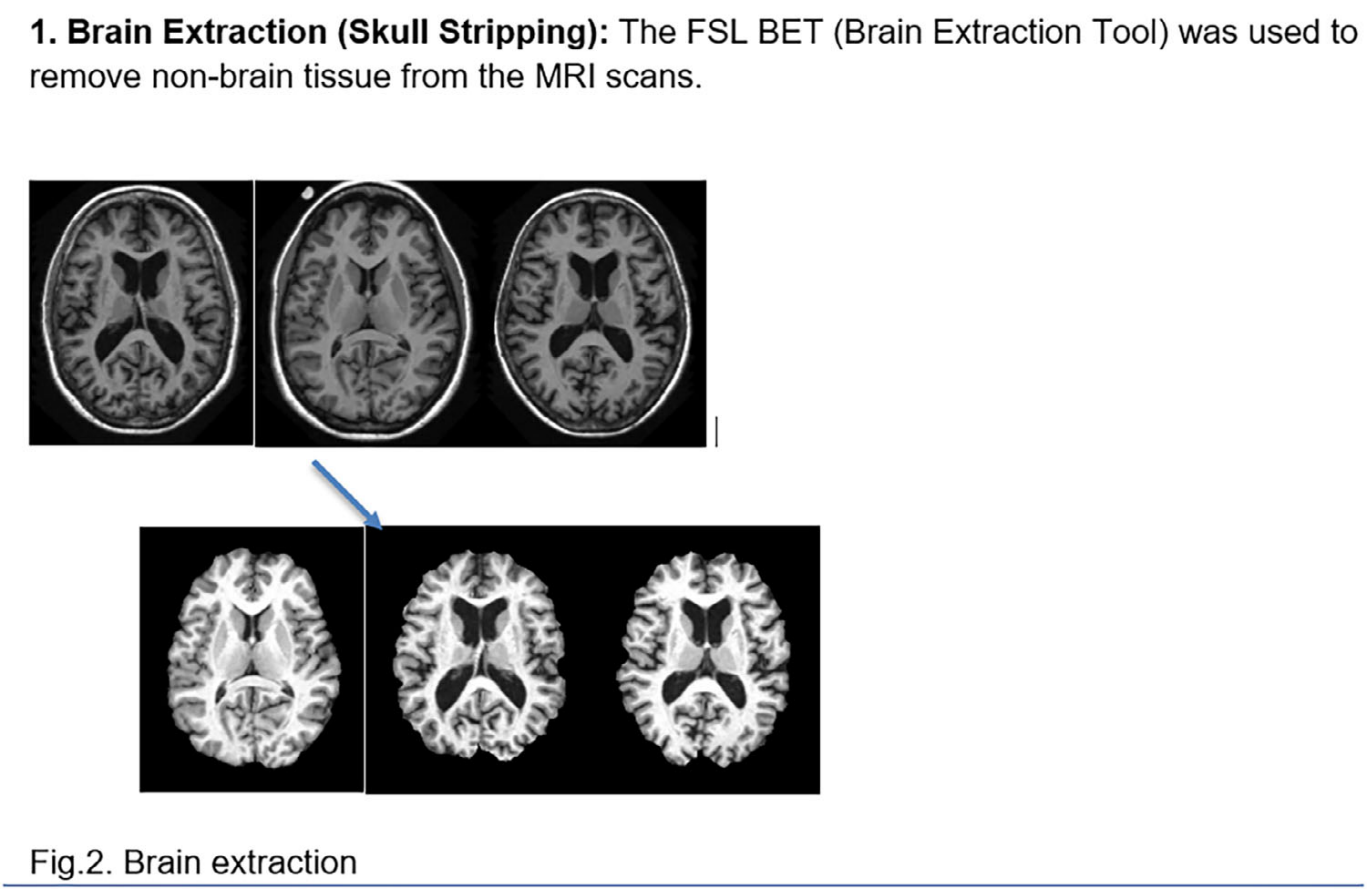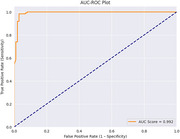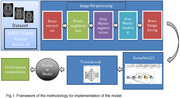# Enhanced Deep Learning Model for Alzheimer's Disease Classification Using Brain MRI: A Nigerian Population Study

**DOI:** 10.1002/alz70856_107749

**Published:** 2026-01-09

**Authors:** Ayokunle Joshua Ola

**Affiliations:** ^1^ University of Ibadan, Ibadan, Oyo, Nigeria

## Abstract

**Background:**

The application of deep learning in Alzheimer's disease (AD) diagnosis has shown promise, but most studies focus on Western populations, potentially limiting their applicability in African contexts. There is a critical need for validated diagnostic tools that account for population‐specific characteristics in neuroimaging analysis.

**Method:**

We developed a transfer learning‐enhanced DenseNet121 architecture for AD classification. The model was initially pre‐trained on the OASIS dataset to learn general AD‐related features, followed by fine‐tuning on a local dataset from the University College Hospital (UCH), Ibadan, Nigeria. The local dataset comprised 140 subjects (63 dementia, 77 non‐dementia cases). Advanced preprocessing techniques, including skull‐stripping, spatial normalization, and grey matter segmentation, were applied to optimize image quality and feature extraction.

**Result:**

Our model achieved exceptional performance metrics with an accuracy of 97.32% and an AUC score of 0.9916. The sensitivity and specificity were 98.37% and 96.04% respectively, with a precision of 96.80% and an F1 score of 97.58%. This performance significantly surpasses previous studies and demonstrates the effectiveness of our transfer learning approach in capturing population‐specific characteristics while maintaining high diagnostic accuracy.

**Conclusion:**

The successful development and validation of our population‐specific model represents a significant advancement in AD diagnosis for African populations. The high performance metrics validate our transfer learning approach and demonstrate that high‐quality AD diagnosis models can be developed for specific populations while leveraging existing datasets for initial feature learning. This work provides a framework for developing locally‐validated diagnostic tools in low‐resource settings.